# Adverse drug events in German hospital routine data: A validation of International Classification of Diseases, 10th revision (ICD-10) diagnostic codes

**DOI:** 10.1371/journal.pone.0187510

**Published:** 2017-11-02

**Authors:** Nils Kuklik, Jürgen Stausberg, Karl-Heinz Jöckel

**Affiliations:** 1 Institute of Medical Informatics, Biometry and Epidemiology (IMIBE), University Hospital Essen, University of Duisburg-Essen, Essen, Germany; 2 Centre for Clinical Trials Essen (ZKSE), Institute of Medical Informatics, Biometry and Epidemiology (IMIBE), University Hospital Essen, University of Duisburg-Essen, Essen, Germany; Universita degli Studi di Firenze, ITALY

## Abstract

**Objective:**

Adverse drug events (ADEs) during hospital stays are a significant problem of healthcare systems. Established monitoring systems lack completeness or are cost intensive. Routinely assigned International Statistical Classification of Diseases and Related Health Problems (ICD) codes could complement existing systems for ADE identification. To analyze the potential of using routine data for ADE detection, the validity of a set of ICD codes was determined focusing on hospital-acquired events.

**Material and methods:**

The study utilized routine data from four German hospitals covering the years 2014 and 2015. A set of ICD, 10^th^ Revision, German Modification (ICD-10-GM) diagnoses coded most frequently in the routine data and identified as codes indicating ADEs was analyzed. Data from psychiatric and psychotherapeutic departments were excluded. Retrospective chart review was performed to calculate positive predictive values (PPV) and sensitivity.

**Results:**

Of 807 reviewed ADE codes, 91.2% (95%-confidence interval: 89.0, 93.1) were identified as disease in the medical records and 65.1% (61.7, 68.3) were confirmed as ADE. For code groups being predominantly hospital-acquired, 78.5% (73.7, 82.9) were confirmed as ADE, ranging from 68.5% to 94.4% dependent on the ICD code. However, sensitivity of inpatient ADEs was relatively low. 49.7% (45.2, 54.2) of 495 identified hospital-acquired ADEs were coded as disease in the routine data, from which a subgroup of 12.1% (9.4, 15.3) was coded as drug-associated disease.

**Conclusions:**

ICD codes from routine data can provide an important contribution to the development and improvement of ADE monitoring systems. Documentation quality is crucial to further increase the PPV, and actions against under-reporting of ADEs in routine data need to be taken.

## Introduction

Adverse drug events (ADEs) are frequently occurring complications in the community and during inpatient treatment. [1] They pose a significant burden on the patient’s recovery and to financial resources of healthcare systems. [2, 3] A prompt identification of such events is a prerequisite in order to avert further damage to third parties by optimizing clinical processes and by increasing the knowledge about specific drugs. Vigilance systems for spontaneous reporting of adverse drug events are established in hospital environments such as Critical Incident Reporting Systems (CIRS), however, they suffer from under-reporting in daily hospital care. [4, 5] Structured chart review is more efficient in identifying ADEs but time and cost intensive. The utilization of routinely collected diagnoses coded by the International Statistical Classification of Diseases and Related Health Problems (ICD) could usefully complement the existing systems in terms of timely detection, accuracy, and completeness, especially when combined with other computerized surveillance systems such as laboratory value triggers or Computerized Physician Order Entry systems.

Diagnoses of inpatients in Germany are coded by the ICD-10-German Modification (ICD-10-GM) system. The codes are part of the hospital routine data that are continuously transmitted to health insurances for reimbursement purposes and annually delivered as a standardized data set to the Institute for the Hospital Remuneration System (InEK). The InEK uses the data for continuous development of the German diagnosis-related groups system. Applying the ICD system for the coding of diagnoses is internationally established and other studies have shown the potential of using ICD codes for the identification of ADEs. [6, 7]

A crucial prerequisite before utilizing ICD coded diagnoses of routine data is a precise recording of the interesting events. In general, false positive as well as false negative diagnoses can be expected. False positive diagnoses are wrongly coded medical conditions that exist in the routine data but are not present in the medical record. False negative diagnoses are conditions that are identified in the medical record but have not been documented in the routine data. So far, in the international literature, most studies analyzed ICD codes with regard to specific diseases without focus on ADEs. [8–11] Little is known about the validity of ICD codes associated with ADEs. A study using data from 2001 and 2003 calculated a positive predictive value (PPV) for ADEs of 64.9% using ICD-9-CM codes. [12] Further international efforts are required to evaluate the quality of ADE-coding ICD diagnoses in hospital routine data and at the same time by using more recent databases. The validity of inpatient ICD-10 codes in Germany in terms of ADEs has not yet been evaluated.

To address this issue, our main objective in this study was to calculate the positive predictive value and the sensitivity of ICD-10-GM coded diagnoses reasonably expected to represent ADEs, with the focus on ADEs that occurred as complication during hospital stay.

## Material and methods

### Definition

An ADE was defined as an injury resulting from medical intervention related to a drug, including either non-preventable harm resulting from appropriate use of medication (adverse drug reaction, ADR) or preventable harm as a result of a medication error. [13–16] The available routine data does not allow a clear differentiation between ADRs and medication errors, thus both event types were subsumed within the definition of an ADE.

### Database

Anonymized routine data was acquired from four full-service hospitals in Germany who provided the data from the calendar years 2014 and 2015. According to the German Hospital Directory, the four hospitals together operated 2,269 beds and treated 109,385 inpatients. Data from all inpatients that were evaluated according to the system of DRGs were included. Data from psychiatric and psychotherapeutic departments were excluded.

Inpatient conditions are coded by ICD-10-GM with one principal diagnosis and several additional diagnoses. The principal diagnosis is defined as “that condition established after study to be chiefly responsible for occasioning the admission of the patient to the hospital for care”, whereas additional diagnoses are defined as “all conditions that coexist at the time of the principal diagnosis, or that develop during the hospital stay” and “requiring diagnostic, therapeutic, supportive, nursing, or monitoring efforts.”. As our focus was on the validation of hospital-acquired ADEs and as by definition hospital-acquired diseases cannot be assigned as principal diagnosis, the analysis was performed with the data set only comprising the additional diagnoses. However, because additional diagnoses include comorbidities as well as complications and no present on admission indicator is available in the German ICD-10-system, the time of event was determined in the course of the study.

### Study population and sampling

#### Positive predictive value

Previously, Stausberg et al. categorized ICD-10-GM codes according to their likelihood of indicating ADEs. [7] It was further reported that the prevalence rate of codes indicating ADEs in Germany is about 4.8%. [17] Based on this classification and the number of codes available from the participating hospitals, ICD-10 codes were selected for evaluation that were classified as codes representing an ADE with high certainty and that were coded most frequently in the routine data of the four hospitals. The codes were grouped into 15 “code groups” (Table 1), for which the PPV was calculated independently. Only codes describing explicit events where included allowing a standardized review, unspecific events such as “T88.7: Unspecified adverse effect of drug or medicament” were excluded. One ICD-10-GM code of an inpatient stay was defined as one observational unit (hereinafter called “case”).

**Table 1:** Selected most frequent codes representing ADEs from ICD-10-GM for positive predictive value evaluation.

*Code group*	*Code description*	*Sample Size*
1: N99.0	Postprocedural renal failure	54
2: D69.52/53	Secondary thrombocytopenia: Heparin-induced thrombocytopenia type I/II	54
3: L27.1	Localized skin eruption due to drugs and medicaments	56
4: F19.2/7	Mental and behavioral disorders due to multiple drug use and use of other psychoactive substances	54
5: F11.2/7	Mental and behavioral disorders due to use of opioids	54
6: G62.0	Drug-induced polyneuropathy	54
7: M81.4	Drug-induced osteoporosis	54
8: F13.2/7	Mental and behavioral disorders due to use of sedatives or hypnotics	48
9: K52.1	Toxic gastroenteritis and colitis	54
10: L27.0	Generalized skin eruption due to drugs and medicaments	54
11: I95.2	Hypotension due to drugs	54
12: D61.1	Drug-induced aplastic anemia	54
13: D70.1-	Drug-induced agranulocytosis and neutropenia	54
14: A04.7	Enterocolitis due to Clostridium difficile	54
15: D69.57/58/59	Other secondary thrombocytopenia	55
Total		807

In German routine data, a PPV of 64% was calculated for nosocomial pneumonia. [18] As in this study specific ICD-10 codes indicating ADEs were selected and because of growing attention in the context of patient safety, a PPV of 80% was expected by assuming a lower 95% confidence limit of 65%. Sample size calculation resulted in a sample size of at least 54 cases per code group.

The cases in each code group have been selected by equal probability random sampling from the additional diagnoses of the routine data. After one code was selected from a hospital stay, all other codes from that inpatient were excluded from following picks to avoid bias and overestimation of the PPV due to oversampling of particularly conspicuous inpatients. The total number of cases selected per hospital was proportional to their number of hospitalizations of 2014 and 2015.

#### Sensitivity

The sensitivity was generally evaluated for hospital-acquired ADEs. In studies on German routine data a sensitivity of 43% (nosocomial pneumonia) and 46% (decubitus) was reported. [18, 19] For sample size calculation, a sensitivity of 50% was expected by assuming a lower 95% confidence limit of 40%, resulting in a sample of 151 ADEs. To increase the probability of identifying events, only patients with a hospital stay of ≥ 10 days were included by assuming an incidence of hospital-acquired ADEs of at least 10%. [17] Medical records of 1510 inpatients were reviewed. The inpatients were selected by equal probability random sampling. Medical charts that were already selected as part of the PPV evaluation were excluded from the sensitivity analysis.

### Chart review

The retrospective review of the medical charts was done by experienced nurses, pharmacists, and hospital medical coders from the participating hospitals after having completed a 1-month training phase.

The information about the ADEs was recorded on standardized forms. For PPV evaluation, individual forms were developed for each code group, comprising detailed information about the event allowing the identification of the code in the medical chart according to standardized medical definitions of the respective disease. The code review was performed on two levels: on a first level it was evaluated if the medical condition described in the ICD-10-GM code can be identified in the medical chart. For example, “D61.1: drug-induced aplastic anemia” was classified as true positive if the disease could be identified, whether or not a drug association was recorded (Level 1: Disease positive). On a second level the cases were validated with regard to the question, whether an ADE can be derived. For example, if the code was “I95.2: Hypotension due to drugs”, hypotension as well as a medication intake associated with the medical condition needed to be identified by chart review (Level 2: ADE positive). In addition, it was determined whether a case was present on admission or hospital-acquired.

To evaluate the sensitivity, a form was created based on the Institute for Healthcare Improvement Trigger Tool for Measuring Adverse Drug Events, a tool developed to detect ADEs by chart review using “triggers” that identify possible ADEs. [20–22] Also ADEs discovered without the presence of a specific trigger were recorded on the form. Review was performed in the hospitals without knowledge of the recorded ICD codes.

### Statistical analysis

PPVs were calculated for each code group by the number of reviewer-confirmed ICD codes divided by the total number of checked ICD codes. The sensitivity was calculated by the number of hospital-acquired ADEs detected by chart review and matching to ICD codes in the routine data divided by the total number of hospital-acquired ADEs detected. Exact 95% confidence intervals (CI) were computed. Analysis was performed using SAS (SAS Institute Inc., Release 9.4).

### Ethics

Sample selection from the routine data was completely performed at the hospital’s site. Only anonymized data was transferred for scientific evaluation. The work was conducted in compliance with ethical standards and the Declaration of Helsinki and according to the requirements of the guidelines of Good Epidemiological Practice [23]. The study was approved by the institutional review board of the university Duisburg-Essen (Ethik-Kommission Universität Duisburg-Essen).

## Results

### PPV

At least 54 cases were selected for each code group according to sample size calculation, except code group eight, for which only 48 cases were available in the routine data. A total of 807 cases were selected for chart review (Table 1).

The results of PPV assessment are summarized in Table 2. Regarding level one evaluation (the medical condition described in the ICD code title was confirmed in the medical record) in 736 cases the medical condition could be identified resulting in a total PPV of 91.2%, with a range of PPVs between 75.9% (D61.1) to 100% (N99.0). About one third of the positive cases were hospital-acquired. Regarding the level two evaluation (the ICD code was identified as ADE in the medical chart) in 525 cases (65.1%) a drug association was identified in the medical record. The individual PPVs range from 5.6% (F11.2/7) to 94.4% (L27.0). The proportion of hospital-acquired ADEs (44%) is higher in comparison to level 1 evaluation.

**Table 2:** Positive predictive value of ICD-10-GM by code group, and number of inpatient events.

*Code group*	*Level*	*N*	*PPV*	*95%-CI*	*Hospital-acquired events [%]*
1: N99.0—Postprocedural renal failure	1: Disease	54/54	100.0	(93.4,100)	41/54 [75.9]
	2: ADE	37/54	68.5	(54.4,80.5)	30/37 [81.1]
2: D69.52/53—Sec. thrombocytopenia: Heparin-induced type I/II	1: Disease	48/54	88.9	(77.4,95.8)	23/48 [47.9]
	2: ADE	38/54	70.4	(56.4,82.0)	22/38 [57.9]
3: L27.1—Localized skin eruption due to drugs and medicaments	1: Disease	51/56	91.1	(80.4,97.0)	36/51 [70.6]
	2: ADE	47/56	83.9	(71.7,92.4)	34/47 [72.3]
4: F19.2/7—Mental and behavioral disorders due to multiple drug use and use of other psychoactive substances	1: Disease	48/54	88.9	(77.4,95.8)	1/48 [2.1]
	2: ADE	10/54	18.5	(9.3,31.4)	1/10 [10.0]
5: F11.2/7—Mental and behavioral disorders due to use of opioids	1: Disease	53/54	98.1	(90.1,100)	0/53 [0]
	2: ADE	3/54	5.6	(1.2,15.4)	0/3 [0]
6: G62.0—Drug-induced polyneuropathy	1: Disease	48/54	88.9	(77.4,95.8)	1/48 [2.1]
	2: ADE	46/54	85.2	(72.9,93.4)	1/46 [2.2]
7: M81.4—Drug-induced osteoporosis	1: Disease	44/54	81.5	(68.6,90.8)	0/44 [0]
	2: ADE	35/54	64.8	(50.6,77.3)	0/35 [0]
8: F13.2/7—Mental and behavioral disorders due to use of sedatives or hypnotics	1: Disease	41/48	85.4	(72.2,93.9)	2/41 [4.9]
	2: ADE	24/48	50.0	(35.2,64.8)	2/24 [8.3]
9: K52.1—Toxic gastroenteritis and colitis	1: Disease	52/54	96.3	(87.3,100)	28/52 [53.8]
	2: ADE	45/54	83.3	(70.7,92.1)	26/45 [57.8]
10: L27.0—Generalized skin eruption due to drugs and medicaments	1: Disease	53/54	98.1	(90.1,100)	41/53 [77.4]
	2: ADE	51/54	94.4	(84.6,98.8)	41/51 [80.4]
11: I95.2—Hypotension due to drugs	1: Disease	50/54	92.6	(82.1,98.0)	28/50 [56.0]
	2: ADE	39/54	72.2	(58.4,83.5)	25/39 [64.1]
12: D61.1—Drug-induced aplastic anemia	1: Disease	41/54	75.9	(62.4,86.5)	3/41 [7.3]
	2: ADE	40/54	74.1	(60.3,85.0)	3/40 [7.5]
13: D70.1—Drug-induced agranulocytosis and neutropenia	1: Disease	48/54	88.9	(77.4,95.8)	17/48 [35.4]
	2: ADE	46/54	85.2	(72.9,93.4)	17/46 [37.0]
14: A04.7—Enterocolitis due to Clostridium difficile	1: Disease	53/54	98.1	(90.1,100)	31/53 [58.5]
	2: ADE	37/54	68.5	(54.4,80.5)	26/37 [70.3]
15: D69.57/58/59—Other secondary thrombocytopenia	1: Disease	52/55	94.5	(84.9,98.9)	5/52 [9.6]
	2: ADE	27/55	49.1	(35.4,62.9)	3/27 [11.1]
Total	1: Disease	736/807	91.2	(89.0,93.1)	257/736 [34.9]
	2: ADE	525/807	65.1	(61.7,68.3)	231/525 [44.0]

To further analyze the influence of the event time point on the PPV, two subsets of code groups with proportions of hospital-acquired events >50% and <50% were formed (Table 3). On both levels of evaluation, the subset “>50%” resulted in a higher PPV compared to the subset “<50%” containing code groups with events predominantly or completely present at admission (Disease: 96% vs. 87.9%; ADE: 78.5% vs. 55.9%).

**Table 3:** Overall positive predictive value of ICD-10-GM by proportion of hospital-acquired events.

*Level*	*Subset*	*N*	*PPV [%]*	*95%-CI*
1: Disease	Code groups > 50% hospital-acquired	313/326	96.0	(93.3,97.9)
	(1: N99.0, 3: L27.1, 9: K52.1, 10: L27.0, 11: I95.2, and 14: A04.7)			
	Code groups < 50% hospital-acquired	423/481	87.9	(84.7,90.7)
	(2: D69.52/53, 4: F19.2/7, 5: F11.2/7, 6: G62.0, 7: M81.4, 8: F13.2/7, 12: D61.1, 13: D70.1, and 15: D69.57/58/59)			
2: ADE	Code groups > 50% hospital-acquired	256/326	78.5	(73.7,82.9)
	(1: N99.0, 3: L27.1, 9: K52.1, 10: L27.0, 11: I95.2, and 14: A04.7)			
	Code groups < 50% hospital-acquired	269/481	55.9	(51.4,60.4)
	(2: D69.52/53, 4: F19.2/7, 5: F11.2/7, 6: G62.0, 7: M81.4, 8: F13.2/7, 12: D61.1, 13: D70.1, and 15: D69.57/58/59)			

### Sensitivity

Of 1510 reviewed inpatient stays, there were 358 stays identified with at least one hospital-acquired ADE (23.7% of 1510 inpatient stays). A total of 495 hospital-acquired ADEs were identified in the 358 medical charts (Table 4). 186 of these events were present in the routine data as ICD-10-GM codes that describe the disease but imply no drug-related causation (sensitivity: 37.6%). Additionally, 60 events were present in the routine data as codes implying a relationship to a drug (sensitivity: 12.1%).

**Table 4:** Sensitivity of ICD-10-GM for adverse drug events.

*Routine Data Status*	*N*	*Sensitivity [%]*	*95%-CI*
Coded as adverse drug event	60/495	12.1	(9.4,15.3)
Coded as event without drug indication	186/495	37.6	(33.3,42.0)
Total	246/495	49.7	(45.2,54.2)

## Discussion

PPV and sensitivity allow conclusions about the quality of routine data. In general, false positive and false negative events are a result of miscoding of a rendered service and documentation quality of the medical records. Our study shows that more than 90% of all physical symptoms within the selected set of ICD-10-GM codes could be confirmed by chart review. About two thirds (65.1%) of the analyzed codes were confirmed as ADEs, which demonstrates the potential of the selected codes as marker for patient harm associated with medication intake.

Not all code groups are equally suitable for ADE identification. In particular, code groups describing mental and behavioral disorders have been shown to be mainly a result of misuse of medication or intake of illegal drugs. Although 44% of all confirmed ADEs were hospital-acquired, diseases such as drug-induced osteoporosis, polyneuropathy, and aplastic anemia were, as expected, almost entirely present at admission. As our focus was on hospital-acquired ADEs, a subgroup was analyzed excluding code groups comprising events predominantly present at admission. In this subgroup, more than 95% of the codes could be linked to a disease in the medical records, and more than three quarters (78.5%) were identified as ADEs, ranging from 68.5% (N99.0, A04.7) to 94.4% (L27.0). One have to note that in case of specific codes such as N99.0 or A04.7, causes other than drugs can also be possible triggers for the disease, resulting in lower PPVs for ADEs. The data indicate that comprehensibility and overall quality of documentation in medical records is likely to be better for hospital-acquired ADEs rather than for ADEs present on admission in respect to the PPV.

Our analysis resulted in a relatively low sensitivity of inpatient ADEs. In total, just a half of the total of 495 hospital-acquired ADEs identified by chart review were actually coded as disease in the routine data and roughly 1 of every 8 ADEs was coded as drug-associated disease.

A study performed in the USA reported a PPV of 64.9% after validating a set of ICD-9-Clinical Modification ADE codes. [12] The same group calculated a sensitivity for inpatient ADEs of 10%. [24] Another study reported a PPV of 94% after validating ICD-10-Australian Modification codes, however, they excluded medication errors and focused on diagnoses with assigned external cause codes. [25] Computerized ADE detection systems were evaluated to perform rather poor, with a range of sensitivity of 40%-94% but showing high false positive rates, with PPVs ranging from 0.9% to 64%. [26]

Different reasons leading to false negatives and false positives can be argued. Hospitals are instructed to code all medical conditions requiring efforts, but economical motivated over- and undercoding has to be discussed. In Germany and many other countries coding is nowadays often carried out by trained medical coders, who are dependent on high quality source documents. Especially when coding ADEs, a relationship of a drug to a particular adverse event might not always be clear without definite statement by a physician, leading to false negative and false positive coded ADEs. Patient information might be split between paper-based and electronic medical records that covers the routine data, resulting in inconsistencies between the record systems. [27] However, false positives and false negatives might balance each other leading to plausible overall frequencies. In some cases difficulties in the review process were observed when a patient was transferred from another hospital, where the initial event occurred, leading to false positive results, possibly due to a lack of information about the case. When reviewing chronic diseases such as osteoporosis, our results indicate that in specific cases a disease was coded in the routine data, although the event was only documented in a previous inpatient stay, but not in the actual medical record of the current stay. Further improvement of the quality of patient documentation in general is a crucial ongoing process. Computer-based medical records are more and more finding their way into hospitals but paper-based documentation is still widespread. In our study, no uniform type of source documents was used even within one hospital, making it difficult to evaluate the impact of documentation method on the PPV. Considering the points outlined above, the reported positive predictive values should be interpreted as lower boundaries, as a missing reference in the medical record to a disease or an associated drug might not always indicate an actual false event in the context of document comprehensibility. As we observed a relatively high rate of events coded as diseases in the routine data which are in fact ADEs, a record linkage with available community-based electronic medication registries could support the ADE surveillance for community-acquired ADEs that are treated in the hospital.

Although this study yields valuable results, several limitations arise by using ICD-10-GM codes for the detection of hospital-acquired ADEs. As the codes themselves allow no differentiation between events present on admission and events acquired during hospital stay, it makes it necessary to estimate the rate of hospital-acquired events before utilizing specific codes for quality management interested in complications of inpatient care. In the contrary, surveillance systems interested in adverse drug events independently of their point of origin benefit from the detection of ambulatory complications with hospital data. Only a limited number of ADE codes were selected for PPV evaluation. However, the transferability of the results to other classified ADE codes [7] might be possible if the time points of the events are evaluated, as all codes with predominantly hospital-acquired event show reasonable PPVs. Although the coding of diseases in hospitals of all inpatients is performed based on standardized methods, an effect of our strategy to select patients with a hospital stay of > 10 days on the sensitivity might be possible but cannot be estimated with our data and should therefore be investigated in future studies.

Like the international version of the ICD-10, within the German system, no consistent process is defined how to code drug interactions. For several medical conditions, diagnosis codes can be used that themselves contain a drug association in their description. In addition, a combination code such as “complication due to drug or medicament” can be used to mark a diagnosis as drug-associated. It is likely that an improvement of consistency within the terminology in future versions encourages hospitals towards a more frequent usage of ADE codes. In the ICD-10-GM, currently no combination codes describing specific medicament classes are available for medicaments in therapeutic use. Also, the combination of medications and illegal drugs within the same code description impedes the selection of suitable codes for ADE identification. It would be worthwhile, taking those issues into account with the upcoming 11^th^ revision of the ICD. [28]

## Conclusion

The PPV of ICD codes analyzed in this study is suitable to detect hospital-acquired ADEs. However, the sensitivity of routine data for hospital-acquired ADEs was found to be low, in many cases ADEs being coded as disease without reference to a medication. Efforts are needed to further improve ADE coding, both with regard to medical record quality as well as in the context of ADE nomenclature in the ICD system. Overall, the results confirm the potential of utilizing ICD-10-GM diagnoses coding for ADEs from administrative routine data in hospital monitoring systems. Coding of diseases by ICD is standard in hospitals and thus easy-to-use for other applications, underlining its great advantage in terms of availability and cost efficiency.

## Supporting information

S1 Dataset Datapoints behind means and confidence interval calculations of PPV and sensitivity. (XLSX)
